# From Injury to Recovery: A Six-Month Longitudinal Analysis of Quality of Life After Adult Trauma

**DOI:** 10.3390/jcm15093295

**Published:** 2026-04-26

**Authors:** João Paulo de Melo Barros, Luís Manuel Mota Sousa, César João Vicente da Fonseca, Josiana de Oliveira Martins Duarte, Ana Lúcia da Silva João

**Affiliations:** 1 Local Health Unit of the Alentejo Coast, 7540-230 Santiago do Cacém, Portugal; josimduarte@gmail.com; 2 Comprehensive Health Research Center (CHRC), 7000-811 Évora, Portugal; luismmsousa@gmail.com (L.M.M.S.); cfonseca@uevora.pt (C.J.V.d.F.); alsjoao@uevora.pt (A.L.d.S.J.); 3 Department of Nursing, Health School of Santarém, 2005-075 Santarém, Portugal; 4 Department of Nursing, Atlantic Health School, 2730-036 Barcarena, Portugal; 5 Department of Nursing, University of Évora, 7000-811 Évora, Portugal

**Keywords:** quality of life, EQ-5D-5L, longitudinal study, adult health, recovery determinants

## Abstract

Traumatic injuries are a major cause of disability in adults, with long-term consequences that extend beyond acute survival. Understanding the longitudinal trajectory of quality of life (QoL) following trauma is essential for optimising recovery pathways. This study aimed to evaluate changes in QoL over a six-month period after injury and to characterise the most affected health domains. **Methods**: A longitudinal observational study was conducted including 136 adult trauma patients. QoL was assessed using the EQ-5D-5L at three time points: retrospectively for the pre-trauma state, and prospectively at one and six months post-injury. Statistical analysis included Paired T-Tests and Cohen’s d to evaluate the significance and magnitude of changes across five dimensions: mobility, self-care, usual activities, pain/discomfort, and anxiety/depression. **Results**: The sample was predominantly male (57.4%), and falls were the most common mechanism of injury (57.4%). One month after trauma, a significant decline was observed across all EQ-5D dimensions (*p* < 0.001), with large effect sizes particularly in usual activities (d = 0.89) and self-care (d = 0.86). At six months, significant improvement was noted in all domains compared to the one-month assessment (*p* < 0.001). However, only mobility returned to pre-trauma levels (*p* = 0.137), while persistent impairments remained in pain/discomfort and anxiety/depression. The EQ-VAS score declined from a pre-trauma mean of 82.74 to 69.00 at one month and partially recovered to 77.29 at six months. Notably, only 15.4% of patients received specialized rehabilitation services. **Conclusions**: Trauma results in a profound immediate reduction in QoL. Although physical mobility tends to recover by six months, functional autonomy and psychological well-being remain compromised. The findings highlight the need for multidisciplinary post-discharge interventions, focusing on pain management and psychological support to bridge the gap in long-term recovery.

## 1. Introduction

Traumatic injuries represent a major global public health challenge, accounting for a substantial proportion of morbidity, disability, and overall disease burden among adult populations [1,2]. Although advances in emergency medicine, surgical techniques, and critical care have significantly improved survival rates, mortality reduction alone is no longer considered the principal indicator of successful trauma management. Contemporary research has progressively shifted its focus toward long-term recovery and the quality of life (QoL) of survivors. Increasingly, trauma is recognised not merely as an acute and isolated clinical event, but as a transformative life experience with enduring physical, psychological, and social consequences [3,4,5,6,7].

Traumatic injuries are associated with significant and multidimensional impairments in QoL, affecting physical, psychological, and social domains. In the physical dimension, individuals frequently experience persistent pain, reduced functional capacity, and limitations in activities of daily living, which may remain evident several months after injury [8]. Psychologically, trauma survivors are at increased risk of anxiety, depression, and post-traumatic stress symptoms, all of which are strongly associated with poorer mental health outcomes and reduced QoL [9]. In addition, these impairments often translate into social consequences, including reduced participation in usual activities and difficulties returning to pre-injury roles, further compromising overall well-being [9].

Evidence from longitudinal studies highlights that the first six months following trauma represent a critical period for recovery and adaptation. Significant improvements in physical health-related QoL are typically observed between the first and sixth month after injury, although recovery remains incomplete for many patients [10]. Notably, even at six months post-injury, overall QoL often remains below population norms, particularly in mental health domains, suggesting persistent psychological burden [9]. Furthermore, only a minority of patients report full recovery at this stage, reinforcing the notion that six months constitutes a key milestone in the transition from acute recovery to longer-term adaptation [10]. Therefore, evaluating outcomes at six months is essential to identify patients at risk of prolonged impairment and to guide targeted multidisciplinary interventions.

The recovery trajectory following trauma is inherently complex and multidimensional, involving the interaction of biological, psychological, and social processes. Evidence suggests that post-traumatic QoL is shaped by a dynamic interplay of risk and protective factors. Poorer outcomes are consistently associated with advanced age, female sex, social isolation, lower educational attainment, and greater injury severity, particularly when accompanied by chronic pain or persistent mobility impairment. Additionally, financial instability resulting from an inability to return to work may exacerbate functional limitations and hinder social reintegration [6,7,11,12]. Conversely, resilience and more favourable recovery trajectories are promoted by protective factors such as robust pre-injury health status, timely and effective acute medical management—including adequate pain control—and early access to comprehensive, multidisciplinary rehabilitation services [6,12,13]. Understanding these determinants is therefore essential for identifying vulnerable individuals and optimising personalised recovery pathways.

Although the factors influencing post-trauma recovery are increasingly recognised, longitudinal evidence describing the evolution of QoL across distinct health domains in the early months following injury remains limited. Existing studies often prioritise short-term functional outcomes or single clinical indicators, which constrains a comprehensive understanding of the multidimensional recovery process. Moreover, inconsistencies between injury severity at presentation and access to post-discharge rehabilitation services suggest potential gaps in continuity of care, with implications for long-term outcomes.

In addition to these limitations, important gaps persist in the longitudinal literature. Many studies rely on global QoL measures, limiting the identification of domain-specific recovery patterns and potentially overlooking persistent impairments, particularly in pain and psychological well-being. Furthermore, the absence of pre-injury baseline assessments in most studies restricts the accurate evaluation of trauma-related changes over time. Finally, the relationship between early recovery trajectories and the utilisation of rehabilitation services remains insufficiently explored in real-world clinical settings.

The present study addresses these gaps through an integrative approach. By combining a retrospective pre-trauma baseline with prospective assessments at one and six months, and by analysing QoL across individual EQ-5D-5L domains with effect size estimation, it provides a more detailed and clinically meaningful characterisation of recovery. In addition, by examining the relationship between persistent impairments and the low uptake of rehabilitation services, the study offers novel insight into gaps in transitional care and unmet recovery needs.

To ensure a systematic theoretical foundation, this study is guided by a hypothesis-driven framework based on the biopsychosocial model. Within this perspective, recovery is conceptualised as a dynamic process shaped by the interaction of biological, psychological, and social factors. Biological factors such as injury severity, anatomical involvement, and comorbidities primarily influence physical functioning (mobility, self-care, and usual activities). Psychological factors, particularly anxiety and depression, reflect emotional responses that may follow distinct trajectories. Social determinants, including socioeconomic conditions and access to rehabilitation, are considered key modifiers of recovery across domains.

Guided by this framework, a longitudinal within-subject design was adopted to examine QoL trajectories across three time points (pre-injury, one month, and six months post-trauma), enabling the identification of differential recovery patterns and persistent impairments.

Accordingly, the study is based on the following hypotheses: (1) trauma leads to a significant deterioration across all QoL domains at one month post-injury compared to baseline; (2) partial recovery occurs by six months, with heterogeneous trajectories, with faster improvement in physical domains than in pain-related and psychological domains; and (3) persistent impairments are associated with limited utilisation of rehabilitation services, reflecting gaps in post-discharge care.

In the present cohort, although 71.3% of patients were triaged as Urgent and 18.4% as Very Urgent in the emergency department, only 15.0% received specialised rehabilitation care. This disparity suggests potential gaps in transitional care pathways and highlights vulnerabilities in post-acute follow-up and continuity of support. The limited integration of structured rehabilitation may partially explain the persistence of functional and psychological impairments observed in trauma survivors.

Accordingly, this longitudinal study followed 136 adult trauma patients from a retrospectively assessed pre-injury baseline through one- and six-month post-injury evaluations. The primary objective was not only to characterise the trajectory of QoL during the first six months after trauma, but also to quantify the magnitude of change across specific EQ-5D-5L domains—mobility, self-care, usual activities, pain/discomfort, and anxiety/depression—and to identify patterns of incomplete recovery over time. By disentangling the differential evolution of physical and psychological dimensions, this study aims to provide clinically meaningful evidence to support risk stratification, optimise transitional care pathways, and inform multidisciplinary interventions designed to bridge the gap between acute survival and sustained functional and psychosocial reintegration.

## 2. Materials and Methods

### 2.1. Study Design and Setting

A longitudinal, observational study was conducted to evaluate the impact of traumatic injuries on the QoL of adult patients. Participants included victims of any kind of trauma admitted to an emergency department of a hospital in the Alentejo region of Portugal, who were followed over a six-month period, with data collection occurring at three distinct intervals: baseline (retrospective pre-trauma), one month post-trauma, and six months post-trauma. The recruitment periods were from 17 March to 21 May 2025. Eligible patients were included in the study during admission or stay in the emergency department, giving written consent for subsequent telephone contact.

A formal a priori sample size calculation was not performed, as the study was based on a consecutive sampling strategy. All eligible adult trauma patients admitted to the emergency department during the predefined recruitment period were considered for inclusion, in line with the real-world and observational nature of the study design. This approach was adopted to enhance external validity and to ensure that the sample reflects routine clinical practice. Only a small number of patients were excluded according to predefined criteria, resulting in a sample that is broadly representative of the target population (*n* = 5).

Although no a priori power analysis was conducted, the final sample size (*n* = 136) is consistent with similar longitudinal studies in trauma settings and is considered adequate to detect clinically meaningful within-subject changes over time. Furthermore, the repeated-measures design increases statistical efficiency by reducing inter-individual variability and enabling paired comparisons across time points. From a post hoc perspective, the available sample size provides sufficient sensitivity to detect moderate effect sizes (Cohen’s d ≥ 0.5) at a significance level of 0.05, supporting the robustness of the statistical analyses.

### 2.2. Inclusion and Exclusion Criteria

The study employed a convenience sampling strategy, with participants recruited consecutively during the predefined data collection period from 17 March to 21 May 2025. The final sample comprised 136 adult trauma patients admitted to the emergency department of a hospital in the Alentejo region of Portugal following a traumatic event. Although this approach was conditioned by the study timeframe and patient flow at the clinical site, it enabled comprehensive longitudinal follow-up of all eligible participants.

To enhance sample homogeneity and minimise potential confounding, specific clinical inclusion and exclusion criteria were defined. Eligible participants were adults with clearly documented traumatic injuries, including blunt trauma, penetrating trauma, or burns, with identifiable anatomical involvement.

Patients were excluded if they presented with severe traumatic brain injury or other injuries associated with significant neurological impairment that could compromise the reliability of self-reported quality of life measures. Additionally, individuals with evidence of active substance abuse (including alcohol or illicit drugs) at the time of injury, as well as those with diagnosed severe psychiatric disorders (e.g., major depressive disorder or psychotic disorders), were excluded due to their potential impact on perceived quality of life and recovery trajectories.

Further exclusion criteria included refusal to participate, inability to provide informed consent, severe cognitive impairment, and inability to complete follow-up assessments. These criteria were established to reduce bias arising from pre-existing or concurrent conditions that could independently influence the study outcomes.

### 2.3. Procedures

Participants were identified by the nursing staff during the clinical triage process within the Emergency Department. At this stage, or during their subsequent stay in the emergency room, the patients or their legal representatives were invited to participate in the study. Written informed consent was obtained from all participants prior to data collection. Data were subsequently retrieved from electronic medical records and supplemented by information provided by patients or their caregivers through structured questionnaire, in person or by telephone. Follow-up assessments at one and six months were conducted via structured telephone interviews by trained research staff. Up to three contact attempts were made for each participant before being considered lost to follow-up.

### 2.4. Main Outcomes and Definitions

QoL served as the primary outcome measure, determined via the EuroQol five-dimension five-level (EQ-5D-5L) questionnaire at one and six months following the traumatic event. The EQ-5D-5L is a validated, generic self-assessment tool comprising two primary sections. The initial section appraises five distinct health domains: mobility, self-care, usual activities, pain/discomfort, and anxiety/depression. For each domain, respondents select one of five levels of functional severity, ranging from ‘no problems’ (level 1) to ‘extreme problems or inability’ (level 5). These dimensional scores may be reported as discrete health profiles or aggregated into a summary index, where higher index values typically reflect a greater degree of health impairment. The secondary component consists of the EQ Visual Analogue Scale (EQ-VAS), a quantitative measure where participants rate their overall health status on a vertical scale from 0 (‘the worst imaginable health state’) to 100 (‘the best imaginable health state’).

### 2.5. Data Collection and Variables/Independent Variables

Sociodemographic and injury-related variables were collected from baseline data, including medical records and information provided by the patient or caregiver.

The independent variables and clinical determinants collected at baseline were categorised into sociodemographic, socioeconomic, clinical domains and trauma-related determinants to evaluate their influence on recovery trajectories. Sociodemographic data included gender (male or female), age (categorised as <65 or ≥65 years), marital status (married/civil union, single, widowed, or divorced), and educational attainment, which ranged from the first cycle of basic education to doctoral levels. Socioeconomic status was assessed through professional situation (active employment or retired) and the presence of reported economic insufficiency. Clinical history and pre-existing comorbidities were documented as binary variables reflecting the presence or absence of conditions such as hypertension, diabetes mellitus, congestive heart failure, and dyslipidaemia, alongside other chronic pathologies including osteoporosis and chronic obstructive pulmonary disease (COPD). Trauma characteristics were meticulously recorded, encompassing the mechanism of injury (closed trauma, penetrating trauma, or burns) and the specific kinematics of the event, such as falls, road traffic accidents involving four-wheel vehicles or motorcycles, and workplace accidents. Finally, clinical severity and management were determined by clinical triage classification (e.g., Very Urgent, Urgent or Non-Urgent) and the specific anatomical regions affected, such as the head, thorax, limbs, or spine. Key management variables, including the requirement for hospitalisation and subsequent access to rehabilitation services, were also documented to assess their impact on long-term outcomes.

### 2.6. Ethical Considerations

All procedures involving human participants were conducted in accordance with the ethical standards of the institutional and national research committee, as well as with the principles of the Declaration of Helsinki. The study was conducted in accordance with the Declaration of Helsinki and approved by the Ethics Committee of Unidade Local de Saúde do Litoral Alentejano, EPE (protocol code 02/2025, approved on 29 January 2025). In addition to obtaining informed consent, specific measures were implemented to ensure data privacy and participant protection throughout the study.

Personal data were handled in compliance with the General Data Protection Regulation (GDPR). All collected information was anonymised and coded using unique identification numbers, ensuring that no directly identifiable data were included in the analytical dataset. Access to the data was restricted to authorised members of the research team and stored in secure, password-protected institutional systems.

During follow-up assessments conducted via telephone, participants’ confidentiality was strictly maintained. Interviews were performed in a private setting, and participants were informed of their right to decline to answer any question or to withdraw from the study at any time without any consequences for their care. No sensitive information beyond the scope of the study objectives was collected. These procedures were implemented to minimise potential risks and ensure the protection of participants’ rights, dignity, and well-being throughout the study.

### 2.7. Statistical Methods

All statistical analyses were performed using IBM SPSS Statistics software (version 30.0). Descriptive statistics were computed for all study variables. Categorical variables were summarised as absolute frequencies and percentages, whereas continuous variables were described using minimum and maximum values, means, and standard deviations (SD).

The EQ-5D-5L dimensions (mobility, self-care, usual activities, pain/discomfort, and anxiety/depression) as well as the EQ-VAS scores were analysed at three time points: retrospectively assessed pre-trauma status, one month post-trauma, and six months post-trauma. Given the repeated-measures design, inferential analyses focused on within-subject comparisons across time. Mean differences between time points (pre-trauma vs. one month; pre-trauma vs. six months; and one month vs. six months) were examined using paired-sample Student’s *t*-tests.

Although the EQ-5D-5L dimensions are ordinal variables with five response levels, they were analysed as continuous variables for inferential purposes. This approach is supported by methodological literature indicating that ordinal scales with five or more ordered categories may reasonably approximate interval-level measurement, particularly in samples of adequate size. Furthermore, the present study included 136 participants, exceeding the conventional threshold (*n* ≥ 30) under which the Central Limit Theorem (CLT) applies. According to the CLT, the sampling distribution of the mean difference tends toward normality as sample size increases, regardless of the underlying distribution of the original variable. Therefore, even if the EQ-5D-5L responses are not strictly normally distributed at the individual level, the distribution of paired mean differences can be assumed to approximate normality in sufficiently large samples.

Additionally, the normality of paired differences was empirically assessed through skewness and kurtosis coefficients, as well as visual inspection of histograms and Q–Q plots, which indicated no substantial deviations from normality. The paired-sample *t*-test is known to be robust to moderate violations of the normality assumption, particularly in balanced repeated-measures designs with sample sizes above 100.

Sensitivity analyses using non-parametric Wilcoxon signed-rank tests yielded consistent results, further supporting the robustness of the findings.

To quantify the magnitude of change over time, effect sizes were calculated using Cohen’s d for paired samples. Effect sizes were interpreted according to conventional thresholds (small: 0.20; moderate: 0.50; large: ≥0.80).

All statistical tests were two-tailed, and statistical significance was established at a *p*-value < 0.05.

Loss to follow-up was minimal in the present study, with an attrition rate of 2.21% between baseline and the one-month assessment, and no additional losses at six months. Given the longitudinal within-subject design, analyses were conducted using a complete-case approach, including only participants with available data at each respective time point.

Due to the very low proportion of missing data, no imputation methods were applied, as their impact on the overall results was considered negligible. Furthermore, the pattern of missingness was assumed to be random, given the absence of systematic differences observed during follow-up procedures. Therefore, an intention-to-treat approach was not applicable, as this study was observational in nature and did not involve group allocation or intervention. This approach is consistent with methodological recommendations for longitudinal observational studies with minimal attrition and supports the validity of the findings.

## 3. Results

Of the initial 136 participants recruited at baseline, 3 were lost to follow-up at one month, and 0 at the six-month mark, resulting in a total attrition rate of 2.21%.

**Figure 1:**
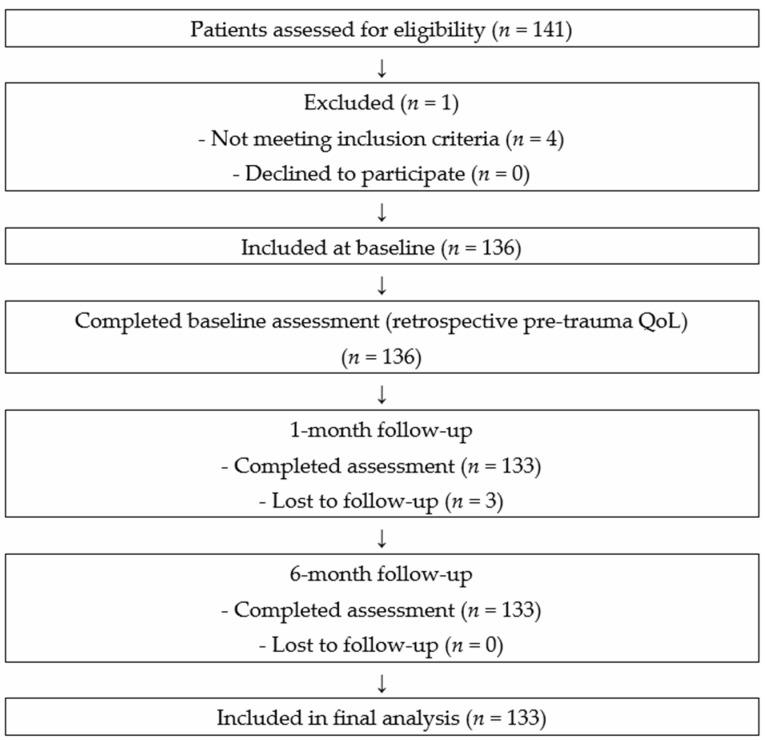
Flow diagram of participant recruitment and follow-up.

The flow of participants throughout the study, from initial recruitment to final follow-up, is illustrated in Figure 1. This flow diagram was developed in accordance with CONSORT recommendations, adapted for observational longitudinal studies, to provide a clear and transparent overview of participant inclusion, exclusion, and retention across the different assessment time points. It details the number of individuals assessed for eligibility, those excluded, and the final sample included at baseline, as well as losses to follow-up at one and six months.

### 3.1. Sociodemographic Characteristics of the Sample

The study cohort comprised 136 participants, of whom 57.4% were male and 42.6% were female. “The age of participants ranged from 18 to 96 years, with a mean age of 56.3 years (SD = 22,24), reflecting a heterogeneous adult population with varying recovery needs following trauma.”

Regarding marital status, the majority of the participants were married or in a civil union (52.2%), followed by those who were single (38.2%). In terms of educational attainment, the most representative groups were those with the first cycle of basic education (29.3%), secondary education (26.3%), and the third cycle of basic education (24.1%). Concerning professional status, 54.1% were in active employment, whilst 39.8% were retired. Furthermore, the vast majority of participants (82.0%) reported no financial insufficiency.

Sociodemographic Characteristics of the Sample are presented in Table 1.

### 3.2. Medical History

Regarding the participants’ clinical history, the most prevalent condition was hypertension (37.0%), followed by dyslipidaemia (21.5%). Diabetes mellitus and congestive heart failure demonstrated an identical prevalence of 12.6%. Issues related to anticoagulation therapy were present in 11.9% of the cohort. The remaining pathologies, specifically osteoporosis, COPD, obesity, anaemia, and stroke, exhibited frequencies of less than 6%.

### 3.3. Triage Classification

Regarding clinical triage, the majority of participants were classified as ‘Urgent’ (71.3%), followed by the ‘Very Urgent’ category (18.4%). Only 10.3% were considered ‘Non Urgent’, reflecting the significant clinical severity of the situations observed within the sample (see Table 2).

### 3.4. Affected Body Region

The anatomical regions most frequently affected were the upper and lower limbs, each accounting for 26.5% of the cases. The head was affected in 19.9% of participants, whilst the thorax was involved in 8.1%. The remaining combinations of body regions exhibited lower frequencies, highlighting the diversity of injuries resulting from trauma.

### 3.5. Mechanism of Injury

The predominant mechanism of injury was blunt trauma, responsible for 87.5% of the cases. Penetrating trauma occurred in 11.0% of participants, whilst burns accounted for only 1.5%, demonstrating a clear prevalence of injuries of contusive origin.

### 3.6. Kinematics of Trauma

Regarding the kinematics of the traumatic event, falls were the most frequent cause, accounting for 57.4% of the cases. Road traffic accidents involving four-wheel vehicles represented 14.7% of the events, whilst motorcycle accidents occurred in 5.9% of the participants. Other less frequent causes included workplace accidents (2.2%), being struck by an object (2.2%), and physical assaults (2.9%), among others (see Table 3).

### 3.7. Hospitalisation and Rehabilitation

Concerning hospital admission at the time of injury, only 18.0% of participants required hospitalisation. In relation to rehabilitation, 15.0% of the cohort attended consultations or underwent rehabilitative treatment, whereas the majority did not seek this type of follow-up care (see Table 4).

### 3.8. Quality of Life (EQ-5D)

Prior to the trauma, the majority of participants reported no problems with mobility (77.4%), self-care (87.2%), or the performance of usual activities (78.9%). In the domain of pain or discomfort, 69.2% indicated an absence of symptoms. With respect to anxiety and depression, 58.6% stated they were asymptomatic, demonstrating a robust functional and emotional health status before the traumatic event (see Table 5).

One month post-trauma, a significant deterioration is observed across all assessed domains. Only 64.7% of participants reported no mobility problems, and 59.4% experienced no difficulties with self-care. Pain or discomfort was present in 74.4% of the sample, primarily of slight to moderate intensity. Furthermore, anxiety and depression levels increased, with only 33.1% of participants reporting an absence of symptoms, as presented in Table 6.

Six months post-trauma, a global improvement is evident in comparison to the outcomes observed at one month. The majority of participants reported no problems regarding mobility (72.9%) or self-care (79.7%). However, limitations in usual activities and symptoms of pain or discomfort persist in a significant proportion of the sample. Similarly, anxiety and depression remain prevalent, albeit with reduced severity (refer to Table 7).

Mean values demonstrate a significant deterioration in QoL at one month post-trauma, followed by a partial recovery at the six-month interval. The mean EQ-VAS score declined from a pre-trauma baseline of 82.74 to 69.00 at one month, subsequently increasing to 77.29 at six months. These results evidence a progressive recovery in perceived health status over the study period, as summarised in Table 8.

### 3.9. Comparison Between Pre-Trauma and One Month Post-Trauma

Mean The comparative analysis between the pre-trauma period and the one-month post-trauma assessment demonstrates a statistically significant deterioration in quality of life across all EQ-5D domains, as well as in the overall health index (EQ-5D VAS). These findings are supported by high significance levels (*p* < 0.001), as presented in Table 9.

In the mobility domain, there was an increase in the mean score from 1.31 (SD = 0.64) to 1.65 (SD = 1.04), corresponding to a mean difference of 0.35 points. This deterioration was statistically significant (t = 4.724; *p* = 0.001) and presented a large effect size (Cohen’s d = 0.84), indicating a clinically relevant impact of trauma on locomotive capacity.

Regarding self-care, the mean increased from 1.19 (SD = 0.54) to 1.70 (SD = 0.97), with a mean difference of 0.51 points. The paired *t*-test revealed statistical significance (t = 6.870; *p* = 0.001) and a large effect size (d = 0.86), suggesting a substantial loss of autonomy in basic care following trauma.

With respect to usual activities, an even more pronounced deterioration was observed, with the mean increasing from 1.30 (SD = 0.65) to 1.92 (SD = 1.00). The mean difference of 0.62 points was statistically significant (t = 8.032; *p* = 0.001) and presented the largest effect size among the functional domains (d = 0.89), reflecting a marked limitation in the ability to perform activities of daily living.

The pain or discomfort domain revealed the most expressive deterioration, with a mean increase of 0.77 points (from 1.36 ± 0.58 to 2.13 ± 0.87). This difference was highly significant (t = 11.994; *p* = 0.001), presenting a large effect size (d = 0.74), indicating that pain constitutes one of the primary factors compromising quality of life in the immediate post-trauma period.

Regarding anxiety or depression, a significant increase in the mean was also observed, from 1.49 (SD = 0.64) to 1.97 (SD = 0.86), with a mean difference of 0.48 points (t = 9.933; *p* < 0.001). The effect size was moderate (d = 0.56), suggesting a relevant emotional impact, though less pronounced than in the physical domains.

Concerning the EQ-5D VAS, a significant decrease in the mean value was observed, from 82.74 (SD = 13.95) to 69.00 (SD = 18.21), corresponding to a mean reduction of 13.74 points. This decrease was statistically significant (t = −10.97; *p* = 0.001) and reflects a substantial deterioration in the global perception of health status following trauma.

### 3.10. Comparison Between the Pre-Trauma Period and 6 Months Post-Trauma

The comparison between the pre-trauma period and the six-month post-trauma assessment reveals that, despite a partial recovery, statistically significant differences persist across several QoL domains (see Table 9). This indicates that the impact of the trauma is not entirely reversible within this temporal interval.

In the mobility domain, the mean difference was minimal (0.05 points) and did not reach statistical significance (t = 1.10; *p* = 0.137), suggesting that mobility generally recovered to levels similar to the pre-trauma state. In contrast, self-care showed a significant increase in the mean score from 1.19 (SD = 0.54) to 1.29 (SD = 0.64), with a mean difference of 0.11 points (t = 3.08; *p* < 0.001). Despite the statistical significance, the effect size was small to moderate (d = 0.39), indicating residual limitations of lesser magnitude.

Usual activities maintained statistically significant differences, with a mean increase of 0.17 points (t = 3.77; *p* < 0.001) and a moderate effect size (d = 0.53), reflecting persistent difficulties in the full resumption of daily routines.

Regarding pain or discomfort, a significant deterioration was observed compared to the pre-trauma period, with a mean difference of 0.23 points (t = 4.90; *p* < 0.001) and a moderate effect size (d = 0.53), indicating that pain remains a relevant symptom at six months.

In the anxiety or depression domain, the mean difference was 0.33 points (t = 6.65; *p* < 0.001), with a moderate effect size (d = 0.57), evidencing the persistence of medium-term emotional impact. Finally, the EQ-5D VAS presented a mean decrease of 5.45 points relative to the pre-trauma period (t = −7.40; *p* < 0.001), suggesting that, despite the improvement observed compared to the one-month post-trauma assessment, the global perception of health status remains inferior to the pre-trauma situation.

### 3.11. Comparison Between One Month and 6 Months Post-Trauma

The analysis of the evolution between one month and six months post-trauma demonstrates a statistically significant improvement across all EQ-5D domains, as well as in the EQ-5D VAS, reflecting a consistent recovery process over time (refer to Table 9).

In the mobility domain, the mean score decreased from 1.65 (SD = 1.04) to 1.35 (SD = 0.63), corresponding to an average improvement of 0.30 points. This difference was statistically significant (t = −5.234; *p* < 0.001), with a moderate effect size (d = 0.66).

Self-care showed a significant improvement of 0.41 points (t = −6.618; *p* < 0.001), with a moderate-to-high effect size (d = 0.71), evidencing an important recovery of functional autonomy.

Regarding usual activities, a mean improvement of 0.44 points was observed (t = −7.085; *p* < 0.001), with a large effect size (d = 0.72), suggesting a progressive resumption of activities of daily living.

Pain or discomfort showed a mean decrease of 0.54 points (t = −10.245; *p* < 0.001), with a moderate effect size (d = 0.61), standing out as one of the domains with the greatest recovery over time.

In relation to anxiety or depression, a significant improvement was observed, albeit of a smaller magnitude, with a mean difference of 0.15 points (t = −3.112; *p* < 0.001) and a moderate effect size (d = 0.56).

Finally, the EQ-5D VAS showed a mean increase of 8.29 points between one and six months post-trauma (t = 10.818; *p* < 0.001), reflecting a significant improvement in the global perception of health status, although it did not fully reach pre-trauma levels.

## 4. Discussion

The analysis of the results underscores the inherent complexity of post-trauma recovery, suggesting that the restoration of biopsychosocial homeostasis is a non-linear and often incomplete process. These findings reinforce the need to move beyond a model centred exclusively on clinical survival towards a more comprehensive approach that prioritises long-term functional outcomes and the role of social determinants of health.

The longitudinal trajectory observed in the present study characterised by a marked deterioration in quality of life at one month, followed by partial recovery at six months is consistent with previous research on post-trauma recovery.

Importantly, the clinical relevance of these changes extends beyond statistical significance and effect size estimates and should be interpreted in light of the minimal clinically important difference (MCID). The MCID represents the smallest change in a patient-reported outcome that is perceived as meaningful by patients and that may justify a modification in clinical management. For the EQ-VAS, previous studies have suggested MCID thresholds generally ranging between 7 and 10 points, although some variability is expected depending on the population and clinical context [14].

In the present study, the substantial decline in EQ-VAS from 82.74 pre-trauma to 69.00 at one month (−13.74 points) exceeds these thresholds, indicating a clinically meaningful deterioration in perceived health status. The subsequent improvement to 77.29 at six months (+8.29 points) also meets the MCID criteria, supporting the clinical significance of recovery over time. However, the remaining difference compared to baseline (−5.45 points) falls below the MCID threshold, suggesting that, despite statistical significance, recovery may not be fully clinically meaningful for a considerable proportion of patients.

This persistent deficit highlights the presence of residual impairment and emphasises the importance of integrating clinical relevance into the interpretation of longitudinal outcomes. In particular, it underscores the need for targeted interventions addressing domains such as pain/discomfort and anxiety/depression, where recovery appears to remain incomplete.

For instance, Nasirian et al. (2020) reported significant improvements in health-related quality of life between discharge, three months, and six months post-injury, although only a minority of patients considered themselves fully recovered at six months [10]. Similarly, large-scale longitudinal analyses have demonstrated a biphasic recovery pattern, with an initial sharp decline followed by gradual improvement that often does not return to pre-injury levels even at 12 months [15].

These findings are further supported by studies showing persistent impairments across multiple EQ-5D domains, particularly in pain/discomfort and usual activities, which tend to recover more slowly than physical mobility [16]. In fact, even long-term follow-up studies indicate that quality of life may remain significantly below population norms years after trauma, especially in more severe cases [17]. The persistence of pain and psychological symptoms observed in our study is therefore aligned with prior evidence highlighting these domains as the most resistant to recovery.

Regarding the magnitude of change, the large effect sizes identified in the early post-trauma phase in the present study are comparable to those reported in previous longitudinal analyses, where clinically meaningful declines in EQ-5D dimensions are observed shortly after injury, followed by moderate improvements over time [18]. However, the persistence of moderate effect sizes at six months in domains such as pain/discomfort and anxiety/depression suggests incomplete recovery, reinforcing the notion that recovery trajectories are heterogeneous and domain-specific.

Overall, our findings corroborate the existing literature by demonstrating that recovery after trauma is not linear but rather characterised by rapid initial deterioration, partial functional recovery, and sustained deficits in specific domains. The relatively low utilisation of rehabilitation services observed in this cohort may further contribute to these differences, as access to post-acute care has been identified as a key determinant of long-term outcomes.

**Dynamics of functional recovery: mobility vs. social participation**

The observed recovery trajectory reveals that the most profound impact on QoL occurs in the first month post-trauma, characterised by a statistically significant deterioration across all EQ-5D domains (*p* < 0.001). This critical period is marked by large effect sizes, particularly in usual activities (d = 0.89) and self-care (d = 0.86), reflecting an abrupt loss of functional autonomy immediately following the traumatic event. This is a phenomenon well-established in the literature and reveals the complex nature of rehabilitation.

Although the ‘mobility’ dimension returned to levels statistically similar to pre-trauma status at six months (*p* = 0.137), ‘usual activities’ remained significantly compromised despite the significant improvement between the first and sixth month (d = 0.72). The ‘usual activities’ domain remains compromised compared to the pre-trauma state (*p* < 0.001). Tefertiller et al. (2023) demonstrate that intensive interventions, such as locomotor training in spinal cord injuries, yield significant gains in mobility (d = 0.40) and occupation, but not necessarily in immediate social or occupational integration[19]. This suggests that the capacity to walk does not automatically translate into community participation, as the latter demands greater physical and cognitive resilience [19].

Longitudinal investigations sustain that a significant proportion of trauma survivors experience states of persistent morbidity, maintaining moderate to severe levels of disability in the long term. Moksnes et al. (2023) report that, twelve months after moderate and severe trauma, half of the patients still present persistent disability (GOSE < 5), evidencing that the return to pre-trauma functional levels is the exception rather than the rule for many [20].

The severe initial impact on ‘self-care’ (d = 0.86) highlights the sudden loss of independence in activities of daily living. Tien et al. (2021) observed in patients with spinal cord injury in Vietnam that injury severity is drastically associated with lower scores in daily activities, perpetuating dependence on third parties and constituting a strong predictor of secondary depression[21].

**The Co-occurrence of Pain, Anxiety and Depression After Trauma**

The findings indicate that pain and depressive symptomatology are among the domains with the lowest rates of recovery over the six-month follow-up period. At six months, nearly half of the sample (48.9%) continued to report persistent pain or discomfort, despite this domain demonstrating one of the highest rates of improvement over time (d = 0.61). Fetz et al. (2023) conducted a retrospective cohort study at a trauma centre in Germany, reporting that up to two-thirds of major trauma patients experience ongoing pain severe enough to compromise quality of life for several years following the initial injury[22].

Similarly, symptoms of anxiety and depression showed notable persistence, with 63.9% of patients still reporting these symptoms at six months [22]. These findings highlight the sustained burden of both physical and psychological sequelae following trauma.

Previous studies, including those by MacGregor et al. (2020) and Lotfalla et al. (2024), have described the frequent co-occurrence of pain, mental health disorders, and physical sequelae as a “clinical triad” associated with poorer quality of life outcomes [12,23]. However, in the present study, although these domains were observed to persist over time, their interrelationship and potential underlying mechanisms were not directly assessed. Therefore, this concept should be interpreted as a theoretical framework derived from existing literature rather than a construct empirically tested within this dataset.

**Discrepancy between Injury Severity and Rehabilitation Provision**

A critical finding of the results is the marked underutilisation of rehabilitation services, at only 15.0%. This occurs despite 89.7% of cases being triaged in the emergency department as ‘Very Urgent’ or ‘Urgent’. This divergence between clinical necessity and the treatment received represents a systemic challenge also identified by Dumovich and Singh (2024) [1]. These authors suggest that restricted access to physiotherapy and psychological support may explain why dimensions such as ‘Pain or Discomfort’ fail to return to baseline levels.

This underutilisation is particularly relevant in the Alentejo region, where geographical dispersion and the centralisation of specialised resources in urban centres may pose barriers to access. In this context, the transition from hospital discharge to community-based recovery may represent a potential gap in continuity of care.

Patients may therefore rely more heavily on informal support from family networks, which, while essential, may not always provide the technical resources required to address complex post-traumatic sequelae. However, the present study does not allow conclusions regarding the impact of follow-up structure on patient outcomes.

The persistence of pain/discomfort and symptoms of anxiety and depression observed in this cohort is consistent with existing literature describing the co-occurrence of physical and psychological sequelae following trauma. These patterns have been conceptualised in previous studies as part of a “clinical triad”, although the interrelationships between these domains were not directly assessed in the present study.

Taken together, these findings may highlight the importance of improving access to coordinated post-discharge care. Approaches such as tele-rehabilitation or integrated community-based pathways may represent potential strategies to enhance continuity of care, although their effectiveness should be evaluated in future studies specifically designed for this purpose.

Given that 54.1% of the sample was within the active working-age population prior to the injury, the low rehabilitation rate may exacerbate the long-term financial impact. Maclennan et al. (2024) emphasise that early multidisciplinary rehabilitation is the primary factor in reducing absenteeism and the indirect costs associated with trauma[11].

The literature substantiates the efficacy of structured, continuous interventions in the treatment of trauma victims. Amarillha-Donoso et al. (2020) demonstrated that post-operative educational programmes significantly improve vitality and social function in elderly patients with hip fractures [24]. Furthermore, Singh and Dwivedi (2024) noted that the use of virtual reality combined with task-based training proved superior to isolated training in enhancing QoL for individuals with spinal cord injuries [25]. This suggests that technological innovation may mitigate the scarcity of traditional resources. In the absence of formal rehabilitation, David et al. (2022) describe recovery as ‘intersocial’, where support from family and neighbours becomes a determinant factor [26]. However, this often places a significant burden on family dynamics and entails considerable financial costs [11].

**Epidemiological Profile, Comorbidities, and Risk Factors**

The sample profile, which is predominantly male (57.4%) and composed of victims of falls (57.4%), aligns with World Health Organization statistics [27].

The high prevalence of hypertension (37.0%) and other pre-existing conditions may serve as factors of resistance to full recovery. Moksnes et al. (2023) reinforce that the presence of pre-injury comorbidities (ASA ≥ 2) is a statistically significant predictor of poorer functional outcomes at 6 and 12 months [20]. Additionally, Samoborec et al. (2020) state that pre-existing mental health issues are among the factors most strongly associated with poor recovery trajectories [28].

**Implications for Clinical Practice**

The present findings support the need to reconsider trauma care beyond the traditional paradigm centred exclusively on acute survival. Within the constraints of an observational design, the results highlight that recovery following trauma is a prolonged and multidimensional process that extends beyond hospital discharge. Accordingly, clinical outcomes may benefit from being evaluated not only in terms of physiological stabilisation or recovery of mobility, but also across broader domains, including functional autonomy, psychological well-being, and social participation over time.

The persistence of impairments in pain/discomfort and anxiety/depression at six months, despite improvements in physical mobility, points to a potentially heterogeneous recovery trajectory. However, the present study does not allow conclusions regarding the mechanisms underlying this pattern. While these findings are consistent with previous literature suggesting that physical and psychological recovery may evolve along distinct trajectories, the relationship between these domains was not directly assessed in this study.

The low utilisation of specialised rehabilitation services observed in this cohort may reflect potential gaps in transitional care. Nevertheless, no causal relationship can be established between access to rehabilitation and patient outcomes based on the current data. These findings should therefore be interpreted as identifying an area of concern that warrants further investigation, rather than as evidence of a direct effect.

From a clinical and health systems perspective, the results align with existing evidence supporting the potential value of coordinated, multidisciplinary approaches to post-trauma care. Such approaches may include structured discharge planning, early referral to rehabilitation services, and follow-up strategies that integrate physical, psychological, and social dimensions of recovery. However, the effectiveness of these strategies cannot be inferred from the present study and should be established in future research.

Overall, the findings should be interpreted within the context of an observational design, which enables the identification of longitudinal patterns but does not support causal inference. While the persistence of impairments in pain/discomfort and anxiety/depression underscores important clinical needs, the mechanisms driving these trajectories remain unclear. Future studies incorporating longitudinal modelling and interventional designs are needed to better understand the relationships between physical recovery, psychological outcomes, and access to rehabilitation services, and to inform evidence-based improvements in trauma care.

## 5. Strengths and Limitations

The present study offers significant contributions to the understanding of the recovery of trauma victims. Its longitudinal design enabled a detailed analysis of individual recovery trajectories, capturing the evolution of QoL across three critical time points. The employment of the EQ-5D-5L instrument, which is extensively validated and internationally recognised, ensures data reliability and facilitates comparability with global literature. Furthermore, by assessing multiple dimensions (both physical and psychosocial), the study offers a holistic perspective that extends beyond clinical survival, identifying specific gaps in post-trauma rehabilitation and emotional support.

However, it is essential to acknowledge the methodological limitations that may influence the interpretation of the findings.

The investigation was conducted exclusively at a single hospital in the Alentejo region of Portugal. The single-centre nature of the study restricts the generalisation (external validity) of the findings to other populations or geographical contexts with differing healthcare infrastructures and rehabilitation systems.

The final sample of 136 adult patients is considered small for an exhaustive analysis of subgroups and independent variables. This sample size reduces the statistical power to detect more subtle differences between specific trauma mechanisms or to perform more robust multivariate analyses regarding the determinants of recovery.

The follow-up period was limited to six months post-trauma. Although this interval captures a critical phase of recovery, it may be insufficient to assess the long-term stabilisation of quality of life or the progression to chronic conditions, particularly in domains such as pain and mental health, which may require a longer timeframe to fully evolve.

Pre-trauma health status was assessed retrospectively, which introduces the potential for recall bias. Patients may overestimate their prior health status due to the impact of the traumatic event or may have difficulty accurately recalling pre-existing functional limitations.

The final sample included 136 patients with complete data across all assessment points. During the follow-up period, three patients were lost due to mortality. Although this attrition rate is low, it may introduce a degree of selection bias, as the exclusion of these cases representing the most severe outcomes could lead to an overestimation of recovery trajectories among the surviving cohort.

In addition, other sources of bias were not formally quantified. Selection bias cannot be excluded, as participation in follow-up assessments may be associated with patient characteristics such as health status, motivation, or access to care. These factors may limit the generalisability of the findings and should be considered when interpreting the results.

## 6. Conclusions

The present longitudinal study demonstrates that trauma precipitates an immediate and multidimensional deterioration in quality of life, extending far beyond the acute clinical phase. Although a statistically significant recovery in physical mobility is observed at six months post-injury, this improvement does not equate to full functional restoration. Domains related to self-care, usual activities, pain/discomfort, and particularly anxiety/depression remain significantly compromised when compared to pre-trauma levels, underscoring the persistent biopsychosocial burden of injury.

The sustained impact of pain and psychological distress highlights the existence of a clinically relevant post-traumatic vulnerability profile, in which physical recovery progresses more rapidly than emotional and functional reintegration. These findings reinforce the notion that survival and motor recovery alone are insufficient indicators of successful outcomes following trauma.

Moreover, the pronounced discrepancy between injury severity at triage and the limited utilisation of specialised rehabilitation services reveals structural gaps in transitional care. The underuse of multidisciplinary rehabilitation constitutes a modifiable barrier to optimal recovery and may contribute to avoidable chronic impairment, prolonged dependency, and reduced social and occupational reintegration.

Collectively, the evidence supports a paradigm shift from an acute, survival-centred model of trauma care towards a longitudinal, patient-centred framework. Early identification of at-risk individuals, systematic follow-up, integrated pain management, and structured psychological support must become standard components of post-discharge pathways. Only through coordinated, multidisciplinary, and continuity-based care strategies will it be possible to effectively bridge the gap between trauma survival and the restoration of full quality of life and societal participation.

## Figures and Tables

**Table 1:** Sociodemographic Characteristics of the Sample.

Variables	Categories	*n*	%
Gender	Female	58	42.6
	Male	78	57.4
Age	<65 years	62	56.4
	≥65 years	48	43.6
Marital Status	Single	52	38.2
	Married/Civil Union	71	52.2
	Widowed	10	7.4
	Divorced/Separated	2	1.5
	1st Cycle	39	29.3
Educational Attainment	2nd Cycle	14	10.5
	3rd Cycle	32	24.1
	Secondary Education	35	26.3
	Bachelor’s Degree	9	6.8
	Master’s Degree	1	0.8
	Doctorate (PhD)	3	2.3
Employed	Yes	72	54.1
	No	61	45.9
Retired	Yes	53	39.8
	No	80	60.2
Financial Insufficiency	Yes	24	18.0
	No	109	82.0

Notes: *n*: absolute frequency; %: percentage. Total sample size N = 136.

**Table 2:** Triage classification of the participants.

Triage	*n*	%
Very Urgent	25	18.4
Urgent	97	71.3
Non Urgent	14	10.3
Total	136	100.0

Notes: *n*: absolute frequency; %: percentage. Total sample size N = 136.

**Table 3:** Observed kinematics of trauma within the sample.

Kinematics of Trauma	N	%
Four-wheel vehicle accident	20	14.7
Motorcycle accident	8	5.9
Falls	78	57.4
Aggression	3	2.2
Workplace accident/personal accident	17	12.5
Others	10	7.4
Total	136	100.0

Notes: *n*: absolute frequency; %: percentage. Total sample size N = 136.

**Table 4:** Hospitalisation and rehabilitation following trauma.

Variables	Categories	*n*	%
Was the patient hospitalised at the time of injury?	Yes	24	18.0
	No	109	82.0
Did the patient receive rehabilitation consultations/treatment?	Yes	20	15.0
	No	113	85.0

Notes: *n*: absolute frequency; %: percentage. Total sample size N = 136.

**Table 5:** Evaluation of pre-trauma quality of life.

	Variables	Categories	*n*	%
Pre-Trauma	Mobility	No problems walking	103	77.4
		Slight problems walking	21	15.8
		Moderate problems walking	7	5.3
		Severe problems walking	2	1.5
	Self-care	No problems washing or dressing	116	87.2
		Slight problems washing or dressing	10	7.5
		Moderate problems washing or dressing	6	4.5
		Severe problems washing or dressing	1	0.8
	Usual activities	No problems performing usual activities	105	78.9
		Slight problems performing usual activities	18	13.5
		Moderate problems performing usual activities	8	6.0
		Severe problems performing usual activities	2	1.5
	Pain or discomfort	No pain or discomfort	92	69.2
		Slight pain or discomfort	34	25.6
		Moderate pain or discomfort	7	5.3
	Anxiety and depression	Not anxious or depressed	78	58.6
		Slightly anxious or depressed	45	33.8
		Moderately anxious or depressed	10	7.5

Notes: *n*: absolute frequency; %: percentage. Total sample size N = 136.

**Table 6:** Evaluation of QoL at one month post-trauma.

	Variables	Categories	N	%
One month post-trauma	Mobility	No problems walking	86	64.7
		Slight problems walking	20	15.0
		Moderate problems walking	17	12.8
		Severe problems walking	7	5.3
		Unable to wal	3	2.3
	Self-care	No problems washing or dressing	79	59.4
		Slight problems washing or dressing	24	18.0
		Moderate problems washing or dressing	21	15.8
		Severe problems washing or dressing	9	6.8
	Usual activities	No problems performing usual activities	59	44.4
		Slight problems performing usual activities	38	28.6
		Moderate problems performing usual activities	25	18.8
		Severe problems performing usual activities	10	7.5
		Unable to perform usual activities	1	0.8
	Pain or discomfort	No pain or discomfort	34	25.6
		Slight pain or discomfort	56	42.1
		Moderate pain or discomfort	35	26.3
		Severe pain or discomfort	8	6.0
	Anxiety and depression	Not anxious or depressed	44	33.1
		Slightly anxious or depressed	56	42.1
		Moderately anxious or depressed	26	19.5
		Severely anxious or depressed	7	5.3

Notes: *n*: absolute frequency; %: percentage. Total sample size N = 136.

**Table 7:** Evaluation of QoL at six months post-trauma.

	Variables	Categories	*n*	%
6 months post-trauma	Mobility	No problems walking	97	72.9
		Slight problems walking	25	18.8
		Moderate problems walking	11	8.3
	Self-care	No problems washing or dressing	106	79.7
		Slight problems washing or dressing	16	12.0
		Moderate problems washing or dressing	10	7.5
		Severe problems washing or dressing	1	0.8
	Usual activities	No problems performing usual activities	87	65.4
		Slight problems performing usual activities	31	23.3
		Moderate problems performing usual activities	13	9.8
		Severe problems performing usual activities	2	1.5
	Pain or discomfort	No pain or discomfort	68	51.1
		Slight pain or discomfort	52	39.1
		Moderate pain or discomfort	13	9.8
	Anxiety and depression	Not anxious or depressed	48	36.1
		Slightly anxious or depressed	63	47.4
		Moderately anxious or depressed	20	15.0
		Severely anxious or depressed	2	1.5

Notes: *n*: absolute frequency; %: percentage. Total sample size N = 136.

**Table 8:** Descriptive statistics for the EQ-5D and EQ-5D VAS domains.

		Minimum	Maximum	Mean	Standard Deviation
Pre-Trauma	Mobility	1.00	4.00	1.31	0.64
	Self-care	1.00	4.00	1.19	0.54
	Usual activities	1.00	4.00	1.30	0.65
	Pain or discomfort	1.00	3.00	1.36	0.58
	Anxiety and depression	1.00	3.00	1.49	0.64
	EQ-5D VAS	50.00	100.00	82.74	13.95
One month post-trauma	Mobility	1.00	5.00	1.65	1.04
	Self-care	1.00	4.00	1.70	0.97
	Usual activities	1.00	5.00	1.92	1.00
	Pain or discomfort	1.00	4.00	2.13	0.87
	Anxiety and depression	1.00	4.00	1.97	0.86
	EQ-5D VAS	10.00	100.00	69.00	18.21
6 months post-trauma	Mobility	1.00	3.00	1.35	0.63
	Self-care	1.00	4.00	1.29	0.64
	Usual activities	1.00	4.00	1.47	0.73
	Pain or discomfort	1.00	3.00	1.59	0.66
	Anxiety and depression	1.00	4.00	1.82	0.74
	EQ-5D VAS	40.00	100.00	77.29	15.27

**Table 9:** Longitudinal Analysis of EQ-5D-5L and EQ-VAS: Mean Differences and Effect Sizes.

EQ-5D Dimensions	T0 vs. T1 (1 Month)		T0 vs. T2 (6 Months)		T1 vs. T2 (6 Months)	
	MD	Cohen’s d	MD	Cohen’s d	MD	Cohen’s d
Mobility	0.35 ***	0.84	0.05	0.47	−0.30 ***	0.66
Self-care	0.51 ***	0.86	0.11 ***	0.39	−0.41 ***	0.71
Usual activities	0.62 ***	0.89	0.17 ***	0.53	−0.44 ***	0.72
Pain or discomfort	0.77 ***	0.74	0.23 ***	0.53	−0.54 ***	0.61
Anxiety or depression	0.48 ***	0.56	0.33 ***	0.57	−0.15 ***	0.56
EQ-5D VAS	−13.74 ***	14.44	−5.45 ***	8.50	8.29 ***	8.83

Notes: T0: Pre-trauma (retrospective); T1: One month post-trauma; T2: Six months post-trauma; MD: Mean Difference; Cohen’s d: Effect size magnitude. Significant levels: *** *p* < 0.001.

## Data Availability

The datasets generated and analysed during the current study are not publicly available due to privacy and ethical restrictions but are available from the corresponding author on reasonable request.

## Abbreviations

The following abbreviations are used in this manuscript:

ASA	American Society of Anesthesiologists
COPD	Chronic Obstructive Pulmonary Disease
EQ-5D-5L	EuroQol five-dimension five-level questionnaire
EQ-VAS	EuroQol Visual Analogue Scale
GOSE	Glasgow Outcome Scale Extended
M	Mean
MD	Mean Difference
QoL	Quality of Life
SD	Standard Deviation
SPSS	Statistical Product and Service Solutions
WHO	World Health Organization
